# Supplementary material for: Oxytocin activity in the paraventricular and supramammillary nuclei of the hypothalamus is essential for social recognition memory in rats
Source: Mol Psychiatry. 2023 Dec 5;29(2):412–24. doi: 10.1038/s41380-023-02336-0 (PMC11116117; doi:10.1038/s41380-023-02336-0)
Supplement: Supplementary file 1 — Supplement data [file 41380_2023_2336_MOESM1_ESM.docx]

**Supplementary materials for**

**Oxytocin Activity in the Paraventricular and Supramammillary Nuclei of the Hypothalamus is Essential for Social Recognition Memory in Rats**

**Authors**

Keerthi Thirtamara Rajamani^1,2,@^, Marie Barbier^1,2^, Arthur Lefevre^3#^, Kristi Niblo^1,2^, Nicholas Cordero^8^, Shai Netser^4^, Valery Grinevich^3^, Shlomo Wagner^4^, Hala Harony-Nicolas^1,2,5,6,7*^

**Affiliations**

^1^Department of Psychiatry and ^2^Seaver Autism Center for Research and Treatment at the Icahn School of Medicine at Mount Sinai, New York, NY, USA, ^3^Department of Neuropeptide Research in Psychiatry, Central Institute of Mental Health, Medical Faculty Mannheim, University of Heidelberg, Mannheim, Germany, ^4^CUNY School of Medicine, The City College of New York, 160 Convent Avenue, NY, USA, ^5^Sagol Department of Neurobiology, University of Haifa, Israel, ^6^Department of Neuroscience, ^7^Friedman Brain Institute, and ^8^Mindich Child Health and Development Institute at the Icahn School of Medicine at Mount Sinai, New York, NY, USA.

# Current Address: Cortical Systems and Behavior Laboratory, University of California San Diego, USA

@ Current Address: Robert Appel Alzheimer’s Disease Research Institute, Weill Cornell Medicine, USA

***Corresponding Author:**

Name: Hala Harony-Nicolas, PhD

Address: 1468 Madison Ave, New York, NY, 10029

Telephone: 212-241-0343

Fax: 212-828-4221

E-mail: [Hala.Harony-Nicolas@mssm.edu](mailto:Hala.Harony-Nicolas@mssm.edu)

This file includes:

Supplementary Materials and Methods

Supplementary Figure 1 to 8

**Supplementary Materials and Methods**

Experimental design

We used a cross-over design wherein the same rat received either 0.9% saline or clozapine N Oxide (CNO)/OXTR antagonist across the testing paradigm. Half of the experimental rats in the cohort received saline and half received CNO/OXTR antagonist and were tested on the short-term social discrimination task to assess short-term SRM. A week later, the rats that previously received saline now received CNO/OXTR antagonist and vice versa and were tested again in the same task. A week later, the same experimental design was repeated but this time, the rats were tested on the long-term social discrimination task to assess long-term SRM. In all experiments, the order of the discrimination tests (i.e. short-term and long-term social recognition memory), was randomized between cohorts.

Histology

Rats were anesthetized using Ketamine (100mg/kg) and Xylazine (13mg/kg). They were then perfused at a rate of 30ml/min with 0.2M Sodium phosphate buffer followed by 4% paraformaldehyde (PFA) at 40ml/min. Brains were immersed in 4% PFA overnight at 4°C, and then in 30% sucrose in 1xPBS for 48h and sectioned on a cryostat (Leica Biosystems, USA).

Immunohistochemistry

To visualize overlap between the DREADD virus and OXT neurons a total of 12 sections spanning the entire PVH were used. Sections were washed blocked and permeabilized for 1h in 5% donkey serum for 1h at room temperature (RT). They were then co-stained with anti-OXT and anti-DsRed antibodies for 24h at 4°C and incubated in donkey anti-mouse IgG 488 and donkey anti-rabbit IgG 594 for 2h at RT and mounted with DAPI containing antifade mounting medium. For immunofluorescence experiments, PVH and SuM sections from an 8 week male SD rat were blocked in 5% donkey serum at RT and co-stained with OXT and calretinin ( or parvalbumin for 24h at 4°C. Sections were then incubated in donkey anti-mouse IgG 488 and donkey anti-rabbit 594 or donkey-anti-goat 594 in for 2h at RT. Similarly, PVH and SuM sections from an OXTp-Venus injected rat were co-stained with anti-GFP and anti-calretinin or anti-parvalbumin antibodies and incubated for 24h at 4°C. This was followed by incubation in donkey anti-chicken IgG 488 and donkey anti-rabbit 594 or donkey anti-goat IgG 594 for 2h at RT. To demonstrate that the retrograde virus targeted the hippocampal CA2 region, hippocampal sections were incubated in anti-Cre and PCP-4. To identify CA2 projecting SuM neurons, SuM sections were incubated with anti-GFP), anti-cre, and anti-calretinin and were processed using similar incubations times as listed above.

To visualize OXT fibers using enzymatic staining, brain sections (40µm) representing the PVH, SON or SuM from an 8 week male SD rat were used. Sections were treated with 3% hydrogen peroxide (H_2_O_2_) followed by 1h incubation in 5% goat serum followed by incubation in anti-OXT for 40h at 4°C. They were then incubated in goat anti mouse HRP 2h at RT, and developed using an ImmPACT diaminobenzidine (DAB) peroxidase substrate. Alternate SuM sections from the same animal were used for staining tissue with 1% cresyl violet. To visualize OXTp-Venus fibers, PVH, SON or SuM sections were treated with 3% H_2_O_2_ incubated in 5% goat serum and then with anti-GFP for 40h at 4°C. Sections were then incubated in the goat anti chicken HRP for 2h at RT and developed using DAB. To examine infusion cannula localization for OXTR antagonist experiments, tissue sections were treated with 1% cresyl violet solution.

Reagents and resources:

| REAGENT OR RESOURCE | SOURCE | IDENTIFIER #/CONCENTRATION | |
| --- | --- | --- | --- |
| Primary Antibodies | | | |
| Mouse monoclonal anti-Oxytocin | A gift from Dr. Harold Gainer | PS 38 | 1:1000 |
| Rabbit polyclonal DsRed | Takara Bio | 632496 | 1:1000 |
| Chicken polyclonal anti-GFP | Thermofisher Scientific | A10262 | 1:1000 |
| Goat polyclonal anti -Parvalbumin | Swant | PVG213 | 1:2000 |
| Rabbit polyclonal anti-Calretinin | Swant | CR7697 | 1:2000 |
| Cre-recombinase | MilliporeSigma | 690503 | 1:2000 |
| PCP-4 | Proteintech | 14705-1-AP | 1:2000 |
| Serum | | | |
| Donkey serum | Jackson Immunoresearch | 017-000-121 | |
| Goat serum | Jackson Immunoresearch | 005-000-121 | |
| Secondary Antibodies | | | |
| Donkey anti-Mouse IgG Alexa Fluor 488 | Thermofisher Scientific | A21202 | 1:1000 |
| Donkey anti-Rabbit IgG Alexa Fluor 594 | Thermofisher Scientific | A21207 | 1:1000 |
| Donkey anti-Goat IgG Alexa Fluor 594 | Thermofisher Scientific | A11058 | 1:1000 |
| Donkey anti-Chicken IgG Alexa Fluor 488 | Jackson Immunoresearch | 703-545-155 | 1:1000 |
| Goat anti-Mouse HRP | Jackson Immunoresearch | 115-035-003 | 1:1000 |
| Goat anti-Chicken HRP | Jackson Immunoresearch | 103-035-155 | 1:1000 |
| Reagents | | | |
| Vectashield anti fade mounting medium with DAPI | Vector Laboratories | H-1200 | |
| Diaminobenzidine substrate peroxidase kit | Vector Laboratories | SK-4105 | |
| Viruses | | | |
| AAV1/2-OXTp-mCherry | Published work^1^ | N/A | |
| AAV1/2-OXTp-hM4Dgi-mCherry | Published work^2^ | N/A | |
| AAV1/2-OXTp-Venus | Published work^1^ | N/A | |
| Ef1a-Cre(AAV Retrograde | Addgene | 55636 | |
| AAV9-EF1a-DIO-eYFP | Addgene | 27056 | |
| Surgical Supplies | | | |
| 20μl NanoFil syringe | World Precision Instruments | NF33BL-2 | |
| 33 gauge needle | Hamilton syringe | HT84853 | |
| Lactated ringer solution | Thermo Scientific | J67572.K2 | |
| EZ wound clips | Stoelting Inc | 59020 | |
| Bone screws | Stoelting Inc | 51457 | |
| Dental cement | Stoelting Inc | 51459 | |
| 1ml BD Luer-Lok syringe | BD Biosciences, | 309328 | |
| Chemicals | | | |
| Clozapine N-Oxide | Cayman Chemicals | 16882 | |
| OXT receptor antagonist (desGly-NH_2_-d(CH_2_)_5_[D-Tyr^2^,Thr^4^]OVT | A gift from by Dr. Mario Manning | N/A | |
| Rat strains | | | |
| Rattus Norvegicus (Sprague Dawley strain) | Charles River Laboratories | N/A | |
| Rattus Norvegicus (Wistar strain) | Charles River Laboratories | N/A | |
| Rattus Norvegicus (Wistar Hannover strain) | Charles River Laboratories | N/A | |
| Fluorescent in-situ hybridization (RNAscope) | | | |
| RNAscope Multiplex Fluorescent Reagent Kit | ACDBio | 323137 | |
| Rat *Oxtr* probe | ACDBio | 483671 | |
| Rat *vglut2* (Slc17a6) probe | ACDBio | 317011 | |
| Rat *vgat1* (Slc32a1) probe | ACDBio | 424541 | |
| Opal 520 | Akoya Biosciences | FP1487001KT | |
| Opal 570 | Akoya Biosciences | FP1488001KT | |
| Opal 690 | Akoya Biosciences | FP1497001KT | |
| Software and algorithms | | | |
| GraphPad Prism 9.0 | GraphPad | <https://www.graphpad.com> | |
| Fiji | ImageJ | <https://imagej.net/software/fiji/> | |
| Adobe Illustrator | Adobe Inc | <https://www.adobe.com/products/illustrator.html> | |
| Matlab | Mathworks Inc | <https://www.mathworks.com/products/matlab.html> | |
| TrackRodent | Custom code | <https://github.com/shainetser/TrackRodent> | |

**References**

1. Knobloch, H.S., Charlet, A., Hoffmann, L.C., Eliava, M., Khrulev, S., Cetin, A.H., Osten, P., Schwarz, M.K., Seeburg, P.H., Stoop, R., and Grinevich, V. (2012). Evoked axonal oxytocin release in the central amygdala attenuates fear response. Neuron *73*, 553-566. 10.1016/j.neuron.2011.11.030.

2. Eliava, M., Melchior, M., Knobloch-Bollmann, H.S., Wahis, J., da Silva Gouveia, M., Tang, Y., Ciobanu, A.C., Triana Del Rio, R., Roth, L.C., Althammer, F., et al. (2016). A New Population of Parvocellular Oxytocin Neurons Controlling Magnocellular Neuron Activity and Inflammatory Pain Processing. Neuron *89*, 1291-1304. 10.1016/j.neuron.2016.01.041.

**Supplementary Figure 1. Chemogenetic silencing of PVH-OXT neurons impairs short-SRM. a.** A schematic of the viral injection **b.** A representative image of PVH-OXT neurons showing AAV1/2-OXT-hM4DGi-mcherry and OXT co-expression in the PVH. **c.** Total investigation time of the novel vs. familiar stimuli during the 2^nd^ encounter for short bouts of interaction (≤6sec) during short-term SRM. No significant differences in the preference for novel over familiar stimuli following Saline or CNO injection (two-way, RM ANOVA, effect of treatment (Saline vs. CNO) x social preference (Familiar vs. Novel) interaction (F_1,26_ = 0.330, *P*=0.570, *ns*))*,* effect of treatment (F_1,26_ = 0.418, *P*=0.523, *ns*), and effect of social preference (F_1,26_ = 2.074, *P*=0.161, *ns*). **d.** Total investigation time of the novel vs. familiar stimuli during the 2^nd^ encounter for long bouts of interaction (≥6sec). There was a significant difference in the preference for novel over familiar stimuli following saline, whereas no difference was observed in preference for novel or familiar stimuli after CNO (treatment x social preference interaction (F_1,26_ = 10.51, ***P*=0.0032)), effect of treatment (F_1,26_ = 5.5, **P*=0.026), and effect of social preference, (F_1,26_ = 26.75, *****P*<0.0001). Post-hoc Sidak multiple comparison test, Saline (Familiar vs. Novel, ^****^*P*<0.0001) and CNO (Fam vs. Nov, *P=*0.424*, ns*)*.* **e.** Investigation time of novel vs familiar stimuli across time following Saline or CNO during short-term SRM. Saline group show consistent preference for novel over familiar stimuli across time (two-way RM ANOVA, time x social preference interaction (F_9,234_ = 2.36, **P=*0.01), effect of social preference (F_1,26_ = 44.71, *****P<0.0001*), and effect of time (F_9,234_ = 1.2, *P=*0.29). **f.** There was no clear preference for familiar or novel stimuli across time following CNO (two-way RM ANOVA, time x social preference interaction (F_9,234_ = 1.500, *P*=0.148, *ns*)), effect of social preference (F_1,26_ = 1.178, *P=*0.28, *ns*), and effect of time (F_9,234_ = 4.135, ***P<0.0001). SRM, Social recognition memory, PVH, paraventricular hypothalamus, OXT, oxytocin, CNO, clozapine-N-oxide. 3V, 3^rd^ ventricle. Data represented as mean ± SEM. Scale bar (100µm). Data represented as Mean ± SEM.

**Supplementary Figure 2. CNO has no impact of SRM and chemogenetic silencing of PVH-OXT neurons does not impair object recognition memory. a.** A representative image showing overlap between AAV1/2-OXTp-mCherry (control) and OXT neurons in the PVH. **b.** Total investigation time of the novel vs. familiar stimuli during the 2^nd^ encounter in rats injected with the AAV1/2-OXTp-mCherry during S-SRM. Both saline and CNO treated rats showed a significant preference for the novel over the familiar social stimuli (two-way repeated measures (RM) ANOVA, social preference (Familiar vs. Novel) x treatment (Saline vs. CNO) interaction (F_1,8_ = 1.311, *P*=0.28, n=5), effect of social preference (F_1,8_ = 75.01, ***P*<0.0001), and effect of treatment (F_1,8_ = 1.15, *P*=0.31). Post-hoc, Sidak multiple comparison test, Saline (Familiar vs. Novel, ****P*=0.0001) and CNO (Familiar vs. Novel, ***P=*0.006). **d.** A Schematic of the novel object recognition paradigm. **e.** Total investigation time of the novel vs. familiar stimuli during the 2^nd^ encounter**.** Saline and CNO injected rats showed significant preference for the novel vs. familiar object (two-way RM ANOVA**,** treatment x social preference (Familiar vs. Novel) interaction, (F_1,14_ = 0.01, *P=*0.91, n=8)), effect of object preference (F_1,14_ = 11.27, ***P<*0.004) and effect of treatment (F_,1,14_ = 0.23, *P=*0.63). Post-hoc Sidak multiple comparison test revealed a significant difference in investigation time between familiar and novel object in both saline and CNO treated conditions saline (Familiar vs. Novel object), **P*=0.01) and CNO (Familiar vs. Novel object, **P=*0.014). **f.** Investigation time of the object stimuli during the 1^st^ encounter. There was no significant difference in the investigation time during the 1^st^ encounter between Saline and CNO injected groups (two-tailed paired student’s *t-*test, t_7_=0.52, *P*=0.61, *ns*). **g.** There was no innate preference for either of the two kinds of objects (Cone vs. Lego) following Saline (left graph) or CNO (right graph) injection (two-tailed unpaired t-test, Cone vs. Lego, Saline, t_7_=0.32, *P*=0.75, *ns,* Cone vs. Lego, CNO, t_8_ = 1.07, *P*=0.31, ns). S-SRM, Short-term Social recognition memory, PVH, paraventricular hypothalamus, OXT, oxytocin, CNO, clozapine-N-oxide. 3V, 3^rd^ ventricle. Data represented as mean ± SEM. Scale bar (100µm). Data represented as Mean ± SEM.

**Supplementary Figure 3. Chemogenetic silencing of PVH-OXT neurons impairs long-term SRM.** Total investigation time of the novel vs. familiar stimuli during the 2^nd^ encounter for short bouts of interaction (≤6secs) during long-term SRM. No significant differences in the preference for novel over familiar stimuli following Saline or CNO (effect of treatment, F_1, 26_ = 0.002, *P*=0.957), effect of social preference (Familiar vs. Novel, F_1, 26_ = 0.01, *P*=0.920, treatment x social preference interaction, F_1, 26_ = 2.396, *P*=0.133). **b.** Total investigation time of the novel vs. familiar stimuli during the 2^nd^ encounter for long bouts of interaction (≥6secs). There was a significant difference in the preference for novel over familiar stimuli following saline, whereas no significant difference in the preference either stimuli was observed following CNO (treatment x social preference interaction, F_1,26_ = 8.08, ***P*=0.008) effect of treatment, (F_1,26_ = 0.011, *P*=0.913), effect of social preference, F_1,26_ = 12.19, ***P*=0.0017). Post-hoc Sidak multiple comparison test, Saline (Familiar vs. Novel, *****P*<0.0001) and CNO (Familiar vs. Novel, *P=*0.957*, ns*)*.* **c.** Investigation time of the novel or familiar stimuli across time following saline or CNO during long-term SRM**.** Saline injected rats showed a clear preference for the novel over the familiar stimuli across time, (two-way RM ANOVA, time x social preference interaction (F_9, 234_ = 0.7475, *P*=0.665, *ns*), effect of time, (F_9, 234_ = 2.038, **P*=0.036), effect of social preference (F_1, 26_ = 28.94, *****P*<0.0001). **d.** The same animals showed no clear preference for the either stimuli across time following CNO injection, (time x social preference (F_9, 234_ = 3.240, ****P=*0.001), effect of social preference (F_1, 26_ = 0.03546, P=0.852, *ns*), effect of time (F_9, 234_ = 2.236, **P*=0.020). L-SRM, Long-term Social recognition memory, Data represented as mean ± SEM.

**Supplementary Figure 4. OXT and Venus positive fibers are distributed through rostral-caudal aspects of the medial and lateral parts of the SuM. a.** Enzymatic labeling for Venus in rats injected with the AAV1/2-OXTp-Venus within the PVH, shows the distribution of Venus-positive fibers in the rostro-caudal aspect of the medial and lateral parts of SuM (Bregma -4.3 to -4.7mm). **b.** Enzymatic labeling for OXT in the SuM of wild type rats shows the distribution of OXT fibers in the rostro-caudal aspect of the medial and lateral parts of the SuM (Bregma -4.3 to -4.7mm). **Left and middle panels** are low (4x) and high magnification (10x) of the SuM, respectively, showing Venus-positive fibers (a) or OXT-positive fibers (b). Right panels are SuM sections stained with cresyl violet to highlight the SuM anatomy**.** Low magnification, scale bar 250µm, high magnification, scale bar 100µm. SuM, supramammillary nucleus, SuMl, lateral supramammillary nucleus, MNu, Mammillary nucleus, pm, principal mammillary tract. OXT, oxytocin.

**Supplementary Figure 5. PVH-OXT projection terminals are localized within the SuM. a. (Left)** A representative PVH section from a wild type rat, which was injected unilaterally with AAV1/2-OXTp-Synaptohpysin-GFP (green), co-stained with an anti-OXT antibody (red) to demonstrated the specificity of the virus**. (Middle)** lower (4x) and (**Right**) higher (10x) magnification of a SuM section showing presence of GPF positive puncta along the length of an OXT-labeled fiber **b.** Immunofluorescent labeling for OXT in the SuM of a wild type rat shows distribution of OXT fibers in the lateral and medial and the rostro-caudal (Bregma -4.3 to -4.7mm) aspect of SuM. (**Left**) A cross section of PVH stained with an anti-OXT antibody. (**Middle**) A cross section of the SuM co-labeled with an anti-OXT antibody to stain OXT fibers and an anti-calretinin antibody to highlight the SuM. (**Right**) A cross section of the SuM co-labeled with an anti-OXT antibody to stain OXT fibers and an anti-parvalbumin to distinguish SuM from the MNu. **c.** (**Left**) A representative PVH section from a wild type rat, which was injected unilaterally with AAV1/2-OXTp-Venus (yellow). (**Middle**) A cross section of the SuM showing the Venus distribution and co-labeled with an anti-calretinin antibody to highlight the SuM. (**Right**) A cross section of the SuM showing the Venus distribution and co-labeled with an anti-parvalbumin to distinguish SuM from the MNu. Low magnification, scale bar 250µm, high magnification, SuMm, medial supramammillary nucleus, 3V, 3^rd^ Ventricle, MNu, Mammillary nucleus, pm, principal mammillary tract. OXT, oxytocin. Scale bar 100µm (**a, b, c**, left, middle and right panels), 20µm (**a**, middle panel) and 50µm (**a**, right panel).

**Supplementary Figure 6. In situ hybridization (RNAscope) using probes for *Oxtr*, *vglut2* and *vgat1* on a SuM section show OXTR distribution within the SuM.** A tiled image of SuM section following hybridization with *Oxtr*, *vgat1* and *vglut2* probes, show the expression pattern of *Oxtr*, *vglut2* and *vgat1* transcripts across the medio-lateral aspect of the SuM. Scale bar 100µm. SuMm, medial supramammillary nucleus, SuMl, lateral supramammillary nucleus MNu, Mammillary nucleus, pm, principal mammillary tract.

**Supplementary Figure 7. Depiction of the drug cannula localizations in all rats that were tested with OXTR antagonist. a.** SuM sections from two independent rats implanted with cannulas to target the SuM are stained with cresyl violet to show an example of an accurate (left) and inaccurate (right) targeting of the SuM, as highlighted with the greed and red arrow, respectively. **b.** A modified Swanson rat atlas image (level 34) indicating correct (green circles) and incorrect (red circle) placement of the cannula tip within the SuM for each of the tested rats within the cohort. -4.4mm and -4.6mm denotes position of the section relative to bregma.

**Supplementary Figure 8. OXTR antagonism in the SuM affects short and Long-term SRM. a.** Total investigation time for novel vs. familiar stimuli during the 2^nd^ encounter for short bouts of interaction (≤6sec) during short-term SRM. There was no significant difference for preference for novel over familiar following saline or OXTR antagonist infusion (treatment x social preference interaction (F_1,16_ = 0.03, *P*=0.86, *ns*), and effect of treatment (F_1,16_ = 0.04, *P*=0.83, *ns*), effect of social preference (Novel vs. Familiar, F_1,16_ = 0.019, *P=*0.89, *ns*) in short bouts. **b.** Total investigation time for the novel vs. familiar stimuli during the 2^nd^ encounter for long bouts (≥6sec). There was a significant difference in the preference for novel over familiar stimuli following saline, whereas the same animals showed no clear preference for novel or familiar stimuli following OXTR antagonist infusion (treatment x social preference interaction (F_1,16_ = 7.41, **P*=0.01), effect of treatment (F_1,18_ = 0.02, *P*=0.88), and effect of social preference (Novel vs. Familiar, F_1,16_ = 3.78, *P=*0.06). Post-hoc, Sidak multiple comparison test, saline (Familiar vs. Novel, ^**^*P*=0.008) and OXTR antagonist (Familiar vs. Novel, *P=*0.54*, ns*). **c.** Investigation time for familiar vs. novel stimuli across time following saline or OXTR antagonist infusion during short-term SRM. Saline infused group showed a clear preference for novel over familiar stimuli across time (two-way RM ANOVA, time x social preference interaction (Familiar vs. Novel) (F_9,144_ = 1.18, *P=*0.30*, ns*), effect of social preference, F_1,16_ = 16.85, ****P=*0.0009), and effect of time (F_5.3,84.83_=3.22, **P=*0.008). **d.** The same animals showed no clear preference for the familiar over the novel stimuli across time following OXTR antagonist infusion (two-way RM ANOVA, time x social preference interaction (F_9,144_ = 0.27, *P*=0.98, *ns*), effect of time (F_4.2,67.7_ = 2.2, *P*=0.07, *ns*), and effect of social preference (Novel vs. Familiar (F_1,16_ = 0.04, *P=*0.83, *ns*). **f.** Total investigation time of the novel vs. familiar stimuli during the 2^nd^ encounter for the short bouts of interaction (≤6sec) during long-term SRM. No significant differences for preference for novel over familiar following saline or OXTR antagonist infusion (treatment x social preference interaction (F_1,20_ = 0.60, *P*=0.44, *ns*), effect of treatment (F_1,20_ = 1.85, *P*=0.18, *ns*), and effect of social preference (Novel vs. Familiar, F_1,20_ = 0.0001, *P=*0.99, *ns*) in short bouts. **g.** Total investigation time of the novel vs. familiar stimuli during the 2^nd^ encounter for the long bouts of interaction (≥6sec). There was a significant preference for novel over familiar stimuli following saline infusion on long bouts, however the same animals after OXTR antagonist infusion did not show a clear preference for either stimuli (treatment x social preference interaction (F_1,20_ = 14.56, ***P*=0.0011), effect of treatment (F_1,20_ = 1.396, *P*=0.25, *ns*), and effect of social preference (F_1,20_ =18.42, ****P=*0.0004). Post-hoc, Sidak multiple comparison test, Saline (Familiar vs. Novel, ^****^*P*<0.0001) and OXTR antagonist (Familiar vs. Novel, *P=*0.76*, ns*). **h.** Investigation time for familiar or novel stimuli across time following saline or OXTR antagonist infusion during long-term SRM (two-way RM ANOVA, time x social preference interaction (F_9,180_ = 1.22, *P=*0.28, *ns*), effect of social preference (F_1,20_ =29.18, *****P<*0.0001), and effect of time (F_5.4,108.4_=0.30, *P=*0.91*, ns*). **i.** The same animals showed no clear preference for the familiar over the novel stimuli across time following OXTR antagonist infusion (two-way RM ANOVA, time x social preference (F_9,180_ = 0.62, *P*=0.77, *ns*), effect of time (F_6.20,124.0_ = 0.76, *P*=0.60, *ns*), and effect of social preference (Novel vs. Familiar, F_1,20_ = 0.4, *P=*0.53, *ns*).
