# Supplementary figures and images for: Oxytocin activity in the paraventricular and supramammillary nuclei of the hypothalamus is essential for social recognition memory in rats
Source: Mol Psychiatry. 2023 Dec 5;29(2):412–24. doi: 10.1038/s41380-023-02336-0 (PMC11116117; doi:10.1038/s41380-023-02336-0)

a.

AAV-OXTp-hM4DG-mCherry

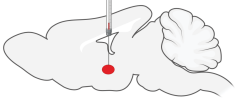

b.

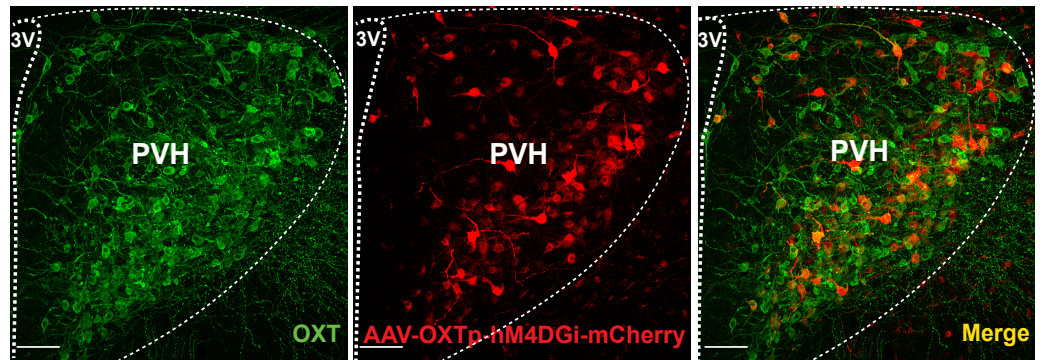

c.

Short bouts ( $\leq 6$ sec)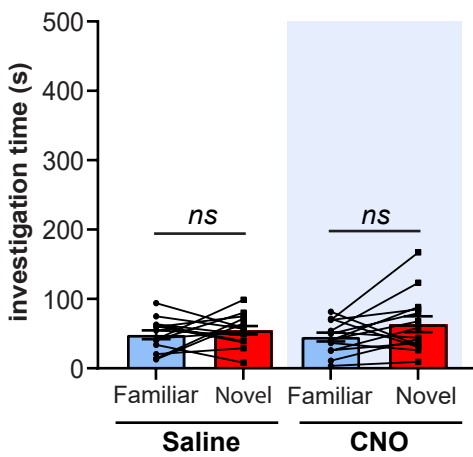

d.

Long bouts ( $\geq 6$ sec)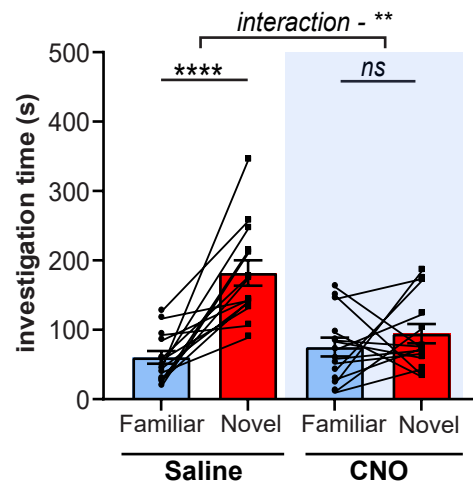

e.

Saline

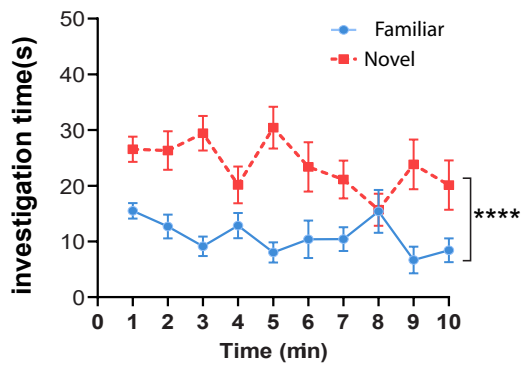

f.

CNO

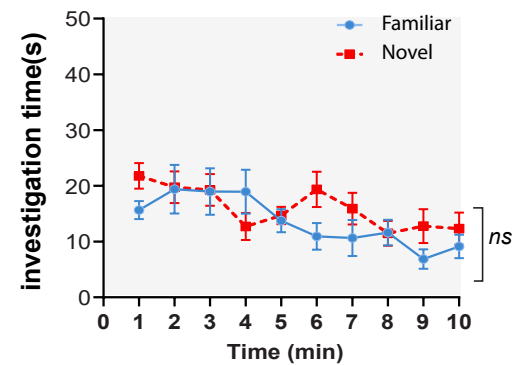

Supplement: Supplementary file 2 — Supplement data Figure 1 [file 41380_2023_2336_MOESM2_ESM.pdf]

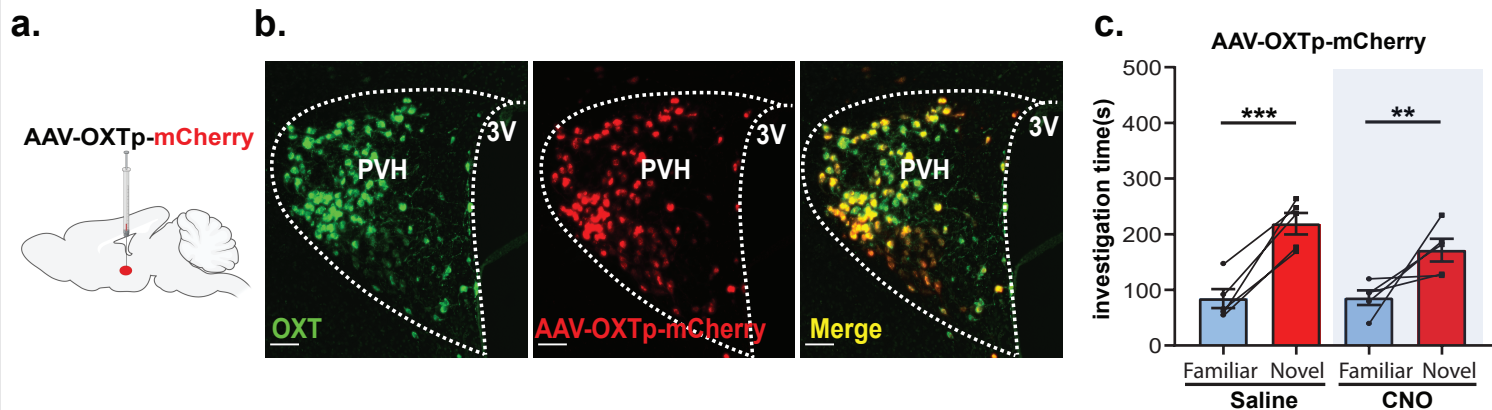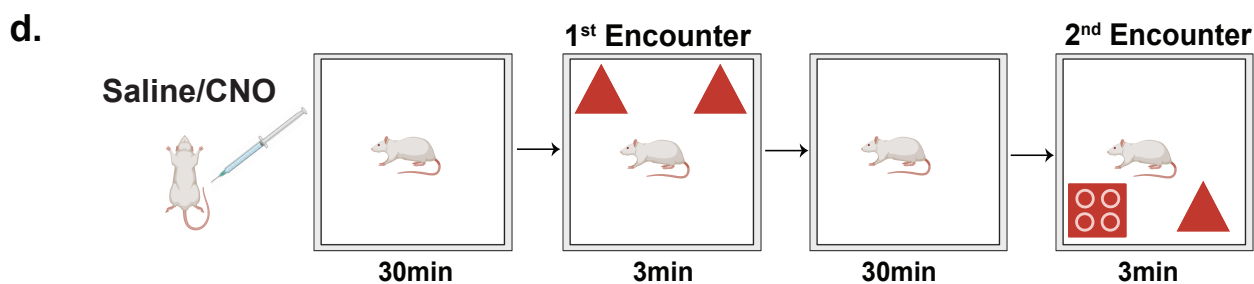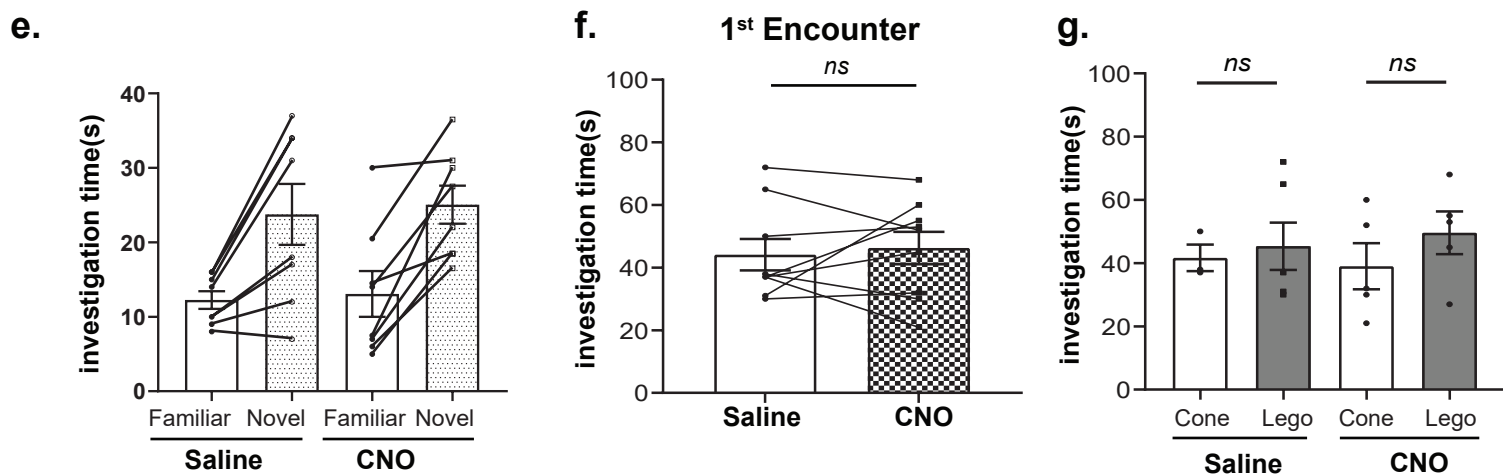

Supplement: Supplementary file 3 — Supplement data Figure 2 [file 41380_2023_2336_MOESM3_ESM.pdf]

**a. Short bouts ( $\leq 6$ sec)**

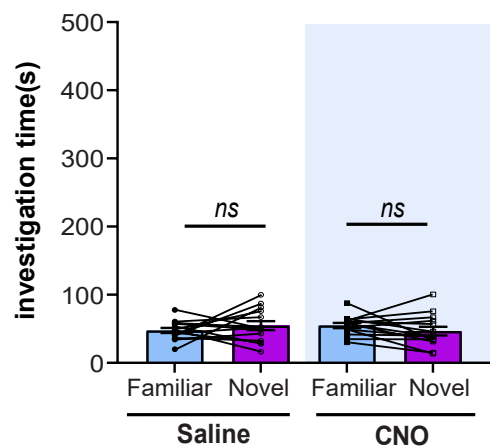

**b. Long bouts ( $\geq 6$ sec)**

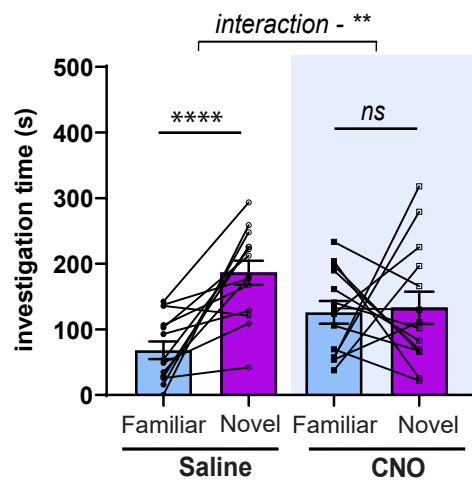

**c. Saline**

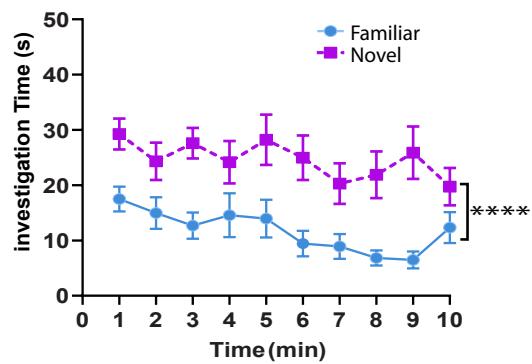

**d. CNO**

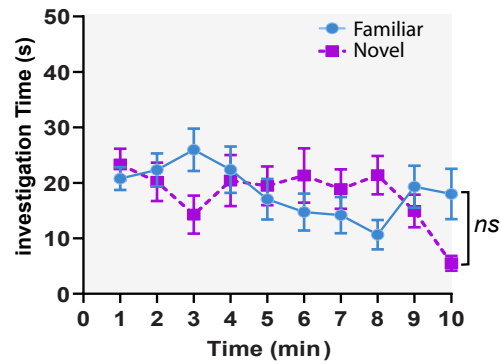

Supplement: Supplementary file 4 — Supplement data Figure 3 [file 41380_2023_2336_MOESM4_ESM.pdf]

a.

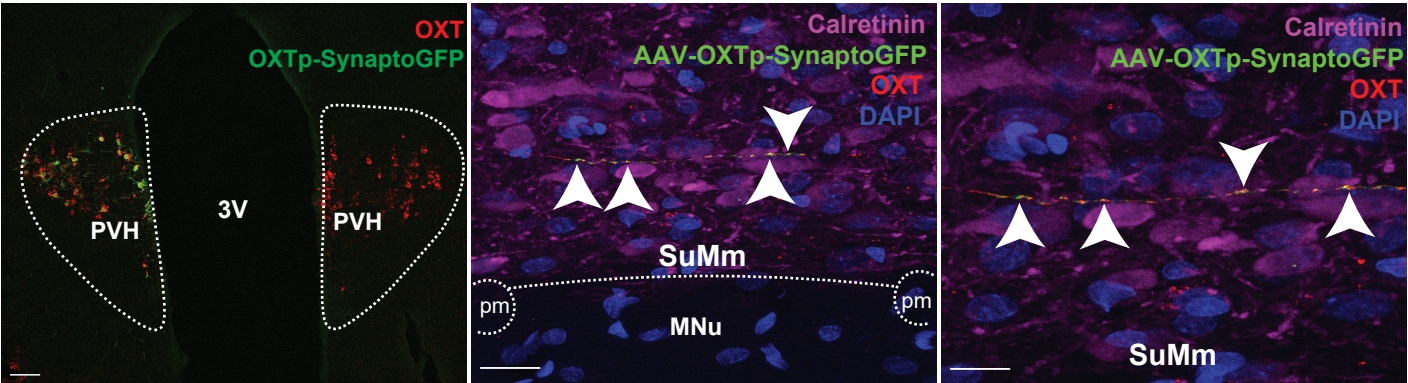

b.

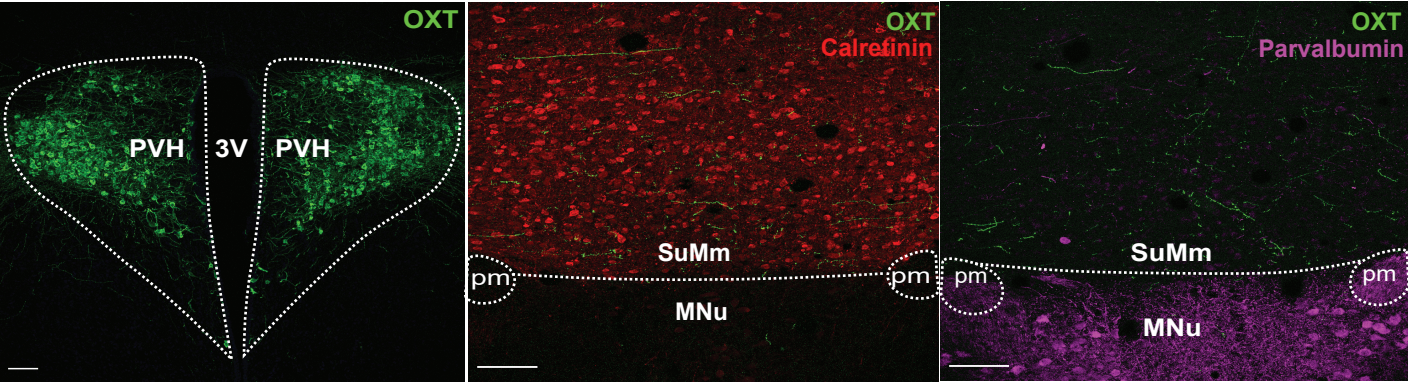

c.

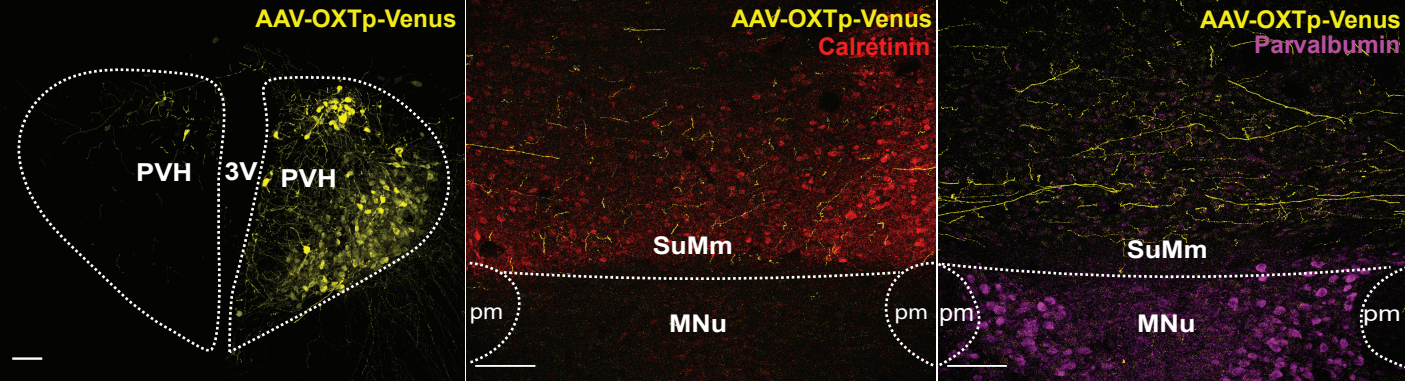

Supplement: Supplementary file 6 — Supplement data Figure 5 [file 41380_2023_2336_MOESM6_ESM.pdf]

**a.**

**Accurate injection**

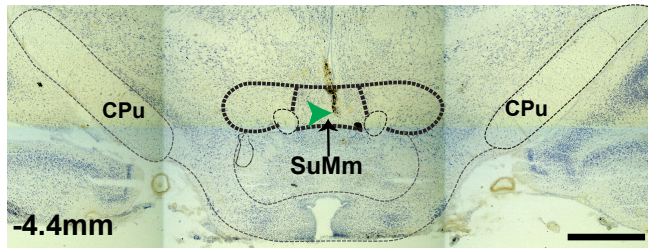

**Miss-injection**

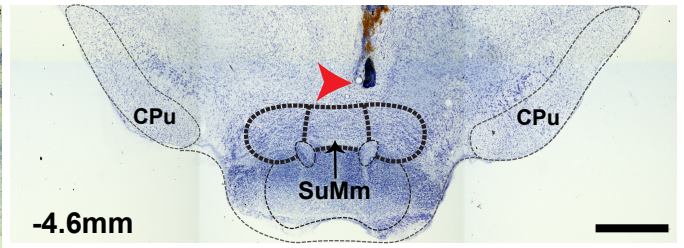

**b.**

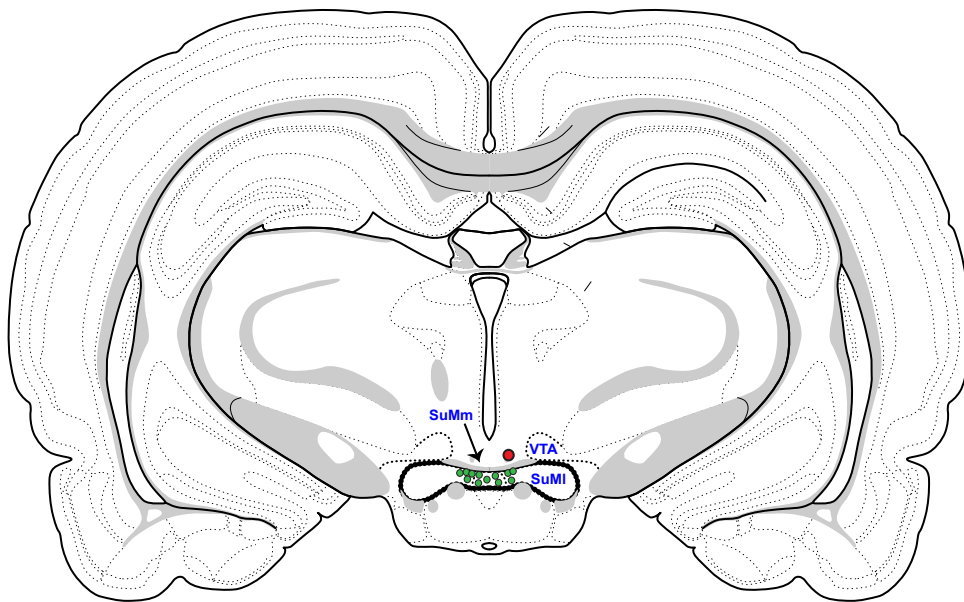

Supplement: Supplementary file 8 — Supplement data Figure 7 [file 41380_2023_2336_MOESM8_ESM.pdf]
