# Supplementary material for: Oxytocin activity in the paraventricular and supramammillary nuclei of the hypothalamus is essential for social recognition memory in rats
Source: Mol Psychiatry. 2023 Dec 5;29(2):412–24. doi: 10.1038/s41380-023-02336-0 (PMC11116117; doi:10.1038/s41380-023-02336-0)
Supplement: Supplementary file 9 — Supplement data Figure 8 [file 41380_2023_2336_MOESM9_ESM.pdf]

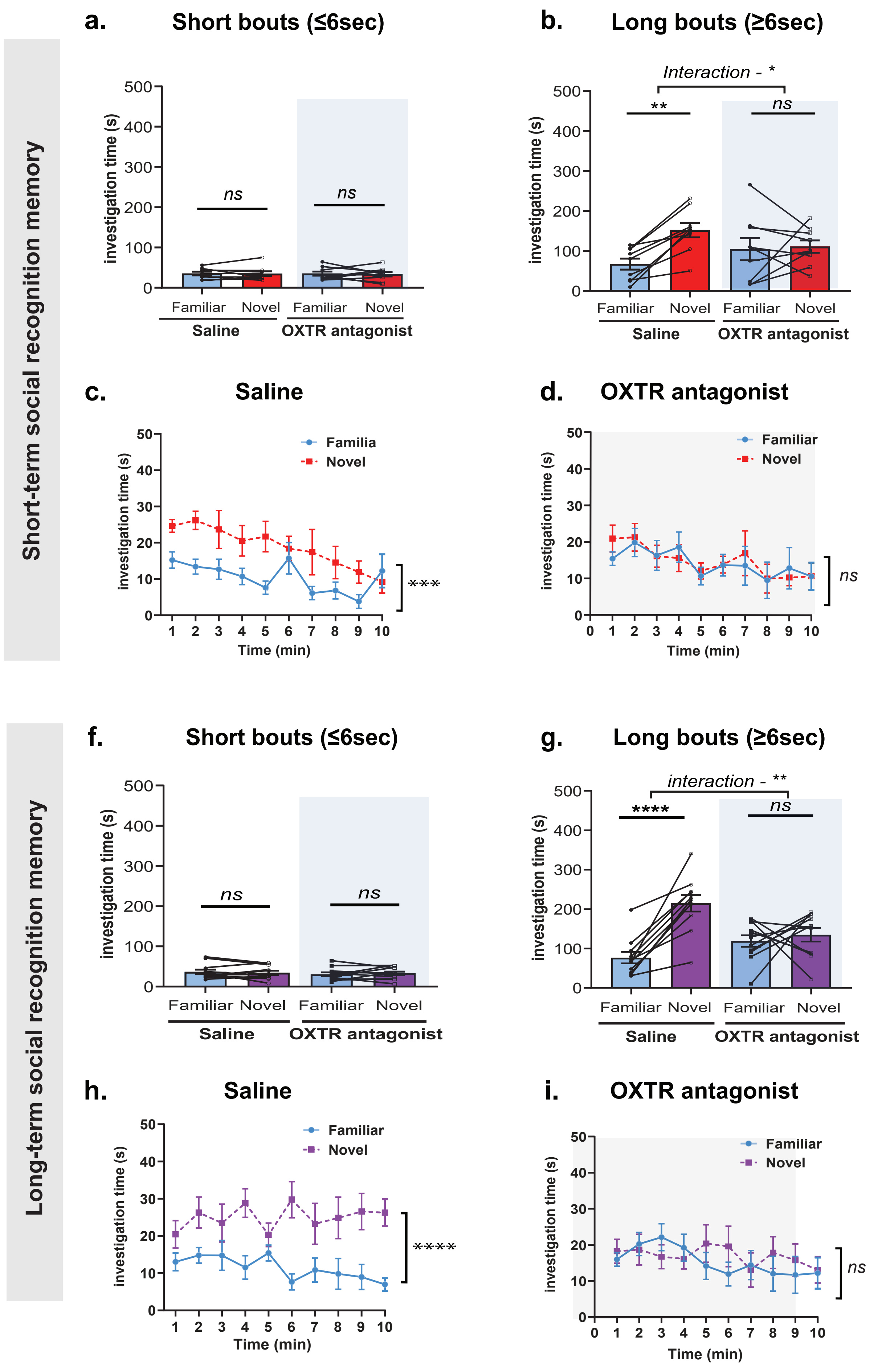
Short-term social recognition memory
a.
Short bouts (≤6sec)
investigation time (s)
ns
ns
Familiar
Novel
Familiar
Novel
Saline
OXTR antagonist
b.
Long bouts (≥6sec)
Interaction - *
**
ns
investigation time (s)
Familiar
Novel
Familiar
Novel
Saline
OXTR antagonist
c.
Saline
Familia
Novel
investigation time (s)
***
Time (min)
d.
OXTR antagonist
Familiar
Novel
investigation time (s)
ns
Time (min)
Long-term social recognition memory
f.
Short bouts (≤6sec)
investigation time (s)
ns
ns
Familiar
Novel
Familiar
Novel
Saline
OXTR antagonist
g.
Long bouts (≥6sec)
interaction - **
****
ns
investigation time (s)
Familiar
Novel
Familiar
Novel
Saline
OXTR antagonist
h.
Saline
Familiar
Novel
investigation time (s)
****
Time (min)
i.
OXTR antagonist
Familiar
Novel
investigation time (s)
ns
Time (min)
