# Supplementary material for: Oxytocin activity in the paraventricular and supramammillary nuclei of the hypothalamus is essential for social recognition memory in rats
Source: Mol Psychiatry. 2023 Dec 5;29(2):412–24. doi: 10.1038/s41380-023-02336-0 (PMC11116117; doi:10.1038/s41380-023-02336-0)
Supplement: Supplementary file 5 — Supplement data Figure 4 [file 41380_2023_2336_MOESM5_ESM.pdf]

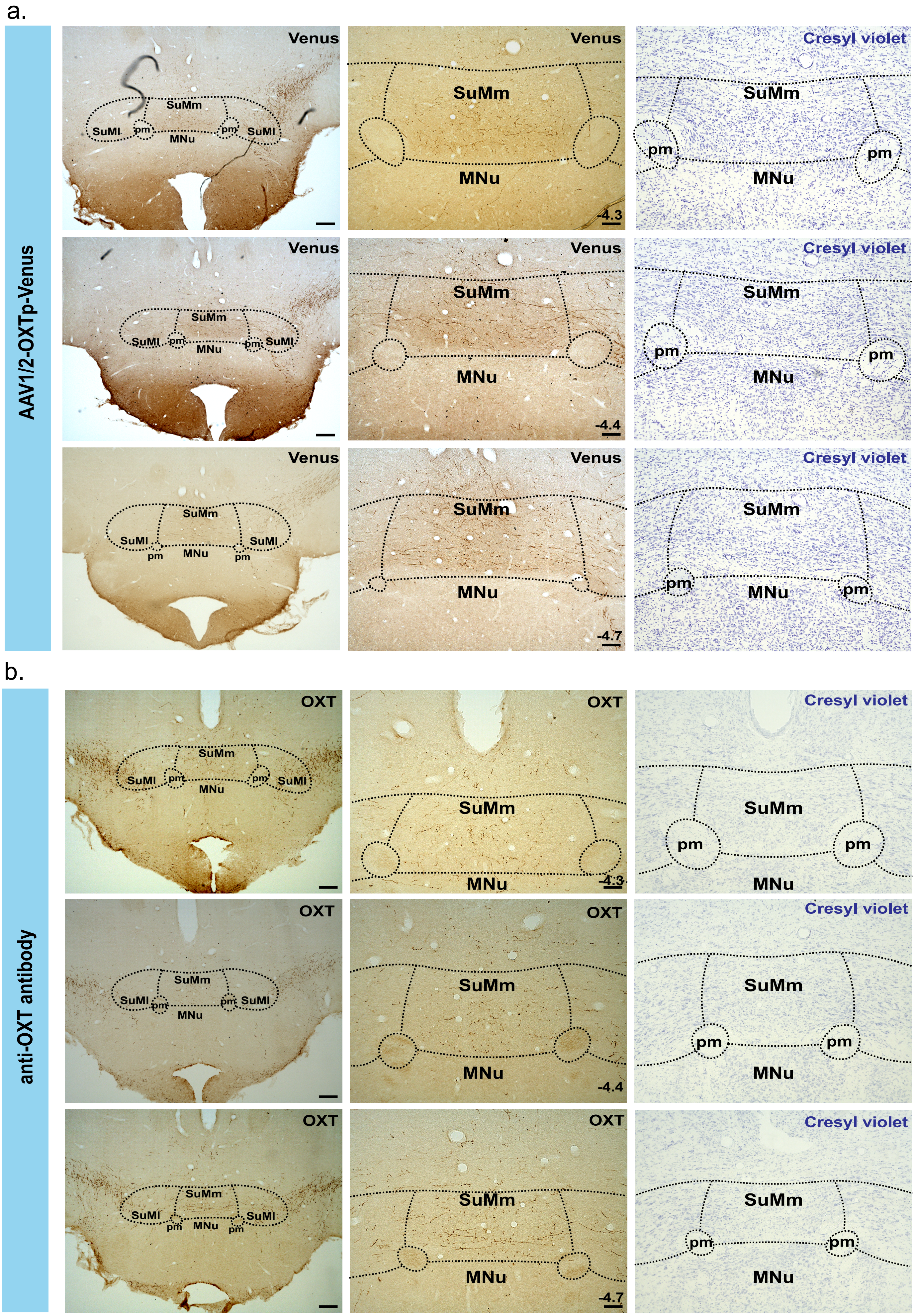
a.
AAV1/2-OXTp-Venus
Venus
Cresyl violet
SuMm
SuMl
pm
MNu
-4.3
-4.4
-4.7
b.
anti-OXT antibody
OXT
Cresyl violet
SuMm
SuMl
pm
MNu
-4.3
-4.4
-4.7
