# Supplementary material for: Oxytocin activity in the paraventricular and supramammillary nuclei of the hypothalamus is essential for social recognition memory in rats
Source: Mol Psychiatry. 2023 Dec 5;29(2):412–24. doi: 10.1038/s41380-023-02336-0 (PMC11116117; doi:10.1038/s41380-023-02336-0)
Supplement: Supplementary file 7 — Supplement data Figure 6 [file 41380_2023_2336_MOESM7_ESM.pdf]

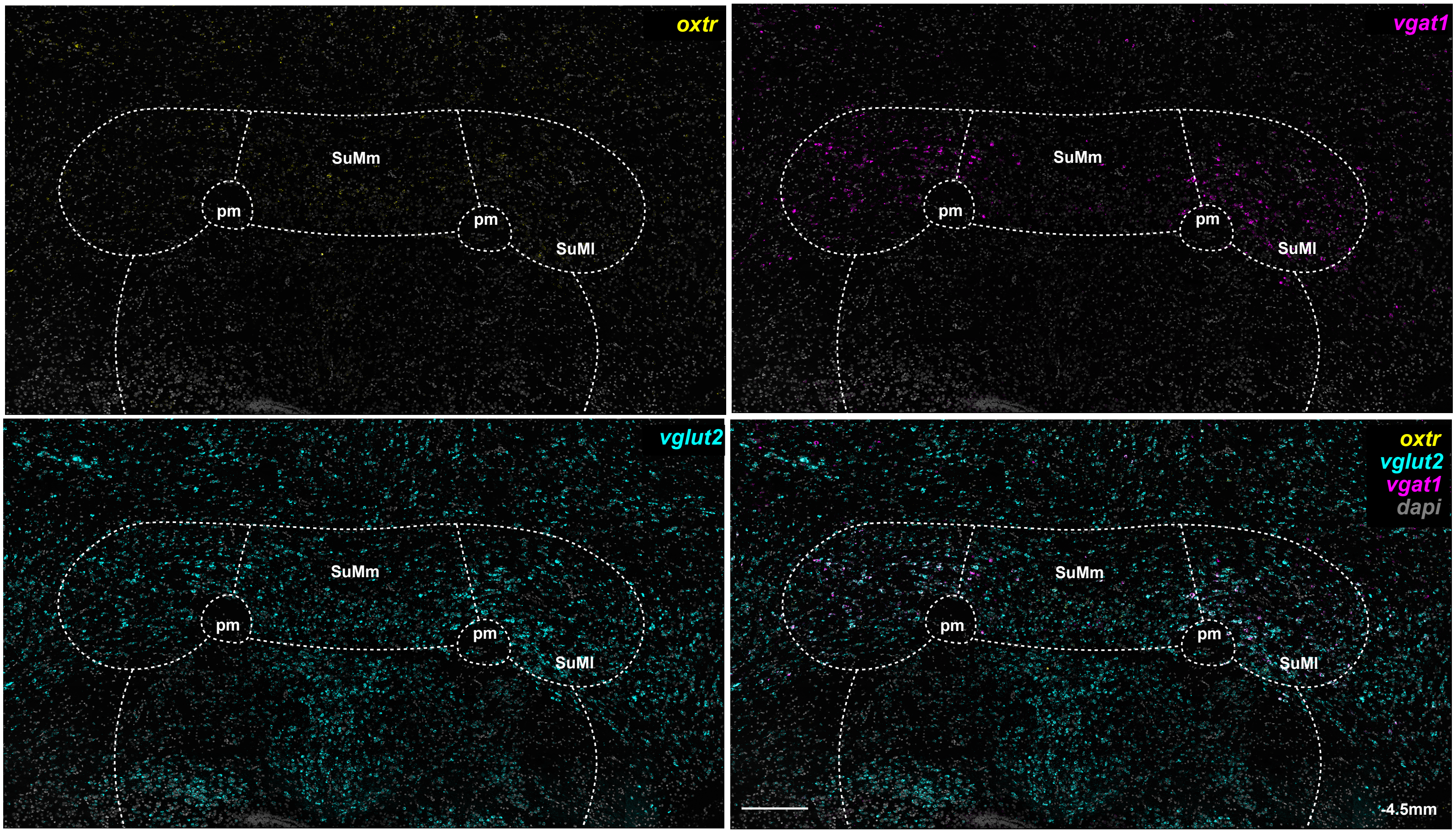
oxtr
SuMm
pm
pm
SuMl
vgat1
SuMm
pm
pm
SuMl
vglut2
SuMm
pm
pm
SuMl
oxtr
vglut2
vgat1
dapi
SuMm
pm
pm
SuMl
-4.5mm
